# Refining AKI Diagnosis: Is There a Return on the Investment?

**DOI:** 10.1016/j.ekir.2026.106463

**Published:** 2026-03-05

**Authors:** Rajit K. Basu

**Affiliations:** 1 Division of Critical Care, Department of Pediatrics, Ann & Robert Lurie Children’s Hospital of Chicago, Northwestern University Feinberg School of Medicine, Chicago, Illinois, USA

See Clinical Research articles on page 4241 and on Article 103772

Nearly 25 years of research has demonstrated the clear negative impact of acute kidney injury (AKI) both in acute and chronically ill patients. Data repeatedly demonstrate a dose-dependent relationship between AKI severity and mortality.^1^, ^2^, ^3^ What remains unclear, however, is if and when windows of therapeutic opportunity exist in the time course of AKI for a given patient. Although the change in serum creatinine (SCr) levels remains the gold standard for diagnosis, evidence strongly suggests that prognosis of the AKI or the AKI-associated patient sequelae is not possible based on SCr alone. In addition, nearly 50% of hospitalized children do not have a previously measured value (requiring imputation for creation of “baseline” values), and small changes in SCr have unclear reliability. In a recent issue of *Kidney International Reports*, 2 studies have attempted to improve AKI diagnosis and prognosis in critically ill children. Radhakrishnan *et al.*^4^ assess the performance of a new AKI staging tool, the pediatric reference change value optimized for AKI in children. Clover-Brown *et al.*^5^ test the ability of the furosemide stress test (FST) to predict severe AKI and the requirement of dialysis (renal replacement therapy [RRT]). Although both were positive studies, an audit of these studies is required to ask questions about the pragmatic, clinical utility of the findings. Is continual refinement of AKI stratification criteria “worth it”? Are prognostic studies of AKI risk making an impact on management decisions? What is the return on investment for the patient for this kind of work?

Different conclusions can be drawn when scrutinizing the work in isolation or as part of the ongoing compendium of AKI epidemiologic work. As “stand-alone” studies, the impact of each may not immediately be perceptible. Radhakrishnan *et al.*^4^ evaluate the pediatric reference change value optimized for AKI in children pediatric-specific AKI classification criteria,^6^ validating the diagnostic criteria, which carries increased specificity of AKI diagnosis with poor outcomes, specifically for smaller children with lower SCr. The value of this study appears modest, an optimization of diagnosis for 5% of the population (reducing incorrect positive diagnosis). The benefit of this small margin could be a reduced burden on nephrology consultation or resource allocation triggered by an AKI diagnosis (important in limited settings). This would have to be balanced by the risk of reduced vigilance for exposure to nephrotoxins and decreased use of kidney-supportive measures (e.g., AKI bundles). Meanwhile, Clover-Brown *et al.*^5^ study the additive predictive benefit of the FST for critically ill children at a high risk of AKI (“positive” renal angina and elevated urinary neutrophil gelatinase associated lipocalin). The authors report that nonresponders to the FST (i.e., reduced urine flow rate) in this population is associated with persistent severe AKI. The additive benefit of this prognostic strategy are modest like in the Radhakrishnan *et al.*^4^ study—given that the predictive capability of FST in the biomarker context was equivocal to the AKI Kidney Disease: Improving Global Outcomes stage at the time of FST (area under the curve: 0.89 vs. 0.90, respectively). The combination of FST and AKI Kidney Disease: Improving Global Outcomes stage increased the area under the curve to 0.94, an improvement with limited effect size. However, in the specific group of patients with stage 3 AKI, nonresponders were more likely to require RRT (49% vs. 6%). This reproduces data from adult patients.^7^ In isolation, though this pair of studies identified significant findings—one diagnostic and the other prognostic—it seems far-fetched either could lead to significant improvements in management (over the currently used Kidney Disease: Improving Global Outcomes staging). Quality of care is not assessed (which could confound patient outcome), and neither study focuses on the use of novel AKI treatments.

Are these studies offering little return on the investment? The other lens to use with these studies is how they integrate into the larger equation of investments made in the AKI space. Improved diagnostics for pediatric AKI are continually required because they open the door to precision medicine. These 2 pediatric studies are key pieces in the story of population enrichment for pediatric AKI management. They are complimentary pieces in the story of enrichment as follows: (i) the Radhakrishnan *et al.*^4^ study augments prognostic enrichment, thereby validating the pediatric reference change value optimized for AKI in children approach to small changes in SCr and the association with disease severity, whereas (ii) the Clover-Brown *et al.*^5^ study augments predictive enrichment, thereby highlighting the treatment response (urine output as the response to the treatment of furosemide used in fluid overloaded patients with AKI). The value of these studies becomes clear when integrated into the evolving picture of AKI management— combining existing tools and constructs, some used in new ways, with a more dynamic and individualized approach to the patient (Figure 1). Heterogeneous patient populations can be separated into targeted populations using the sequential use of these enrichment techniques and other risk stratification and biomarker strategies (e.g., renal angina index scoring). Combining the sensitivity of improved diagnostic markers with the specificity of improved prognostic markers separates a given population of children with AKI into clinically meaningful subpopulations. Importantly, the subpopulation separation may be identifying specific AKI phenotypes with a poor prognosis or, conversely, those amenable to a given treatment (Figure 1). The question of added value depends on the clinician response,that is, how does management change if enrichment techniques are used in clinical care? The answers could be quite simple to complex. Earlier windows of therapeutic opportunity are identifiable—pathophysiological changes in the kidney that occur in acute illness can be detected by some novel biomarkers, detectable hours or even days before creatinine level rise is detected or urine output criteria are met. Functional glomerular stress, deemed “subclinical” AKI, is distinguishable from tubular damage AKI by combining functional and tubular damage markers together. Studies such as those done by Radhakrishnan *et al.*^4^ increase the ability to study the long-term consequences of AKI. We do not yet know the long-term cardiometabolic health sequelae in adults who had AKI as children. Studies such as those done by Clover-Brown *et al.*^5^ increase the ability to understand the risk-benefit of therapeutic interventions. For example, though adult RRT initiation trials did not demonstrate a benefit for early initiation,^8^ a population enrichment-based approach was not used. Would early initiation of RRT (by study protocol) have benefited patients who might have been FST responders? This appears to be the case as shown in a preliminary study in pedatric patients. Fluid management for a specific subset of children was identified by a risk stratification system (renal angina index) tool for population enrichment followed by directed biomarker testing.^9^ A clinical decision support algorithm for fluid management was then integrated and the result was improved patient based, ICU based, and resource based outcomes for these children compared to a historical matched cohort without the enrichment strategy.

**Figure 1:**
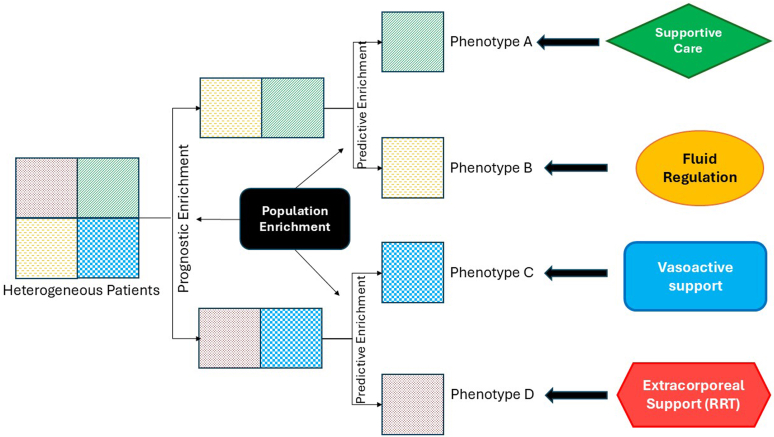
Precision medicine in acute kidney injury: Through a sequential approach to risk stratification and biomarker adjudication, distinct subpopulations of acute kidney injury may be elucidated. Each functionally responsive to specific management. RRT, renal replacement therapy.

As the third decade of intense study on AKI nears completion, critical care nephrology is at last beginning to integrate prognostic and predictive enrichment techniques for precision AKI management. The first decade focused on taxonomy and epidemiology, the second on novel biomarkers. The third has been on learning how to integrate data into clinical decision support tools. These clinical decision support–based approaches are dependent on continued foundational work refining both diagnostic and prognostic markers and will directly impact patient outcome. The return on investment remains an unrealized equation; however, the verdict may be closer than realized.

## Disclosure

The author discloses financial relationships relevant to this editorial; BioPorto Diagnostics (consultancy) and Vantive (Speaker’s Bureau).
